# New Drug for Carcinoma

**Published:** 1913-01

**Authors:** 


NEW DRUG FOR CARCINOMA.—Letulle (*Bulletin de L'Academie de Medecine*) presents a report concerning a compound of arsenic and phosphorus with albumin prepared by Gnezda and designated as arphoalin. It is a brown, tasteless, insoluble powder, and contains 6.3 milligrammes of arsenic and about 6 milligrammes of phosphorus to every gramme of albumin. Applied locally to ulcerated or suppurating tumors it promptly arrests hemorrhage and pus formation and removes odor. Microscopically, Gnezda found that epitheliomatous cells under its influence underwent rapid fatty degeneration, while the stroma soon showed partial necrosis, with gradual healing of the lesion thereafter. Internally, the dose used was 0.25 gramme, an hour after one, two, or even three meals, according to the patient's general condition. Under this treatment rapid necrotic softening of a carcinoma of the neck in a man was noted as well as healing of a recurrent carcinoma of the left cheek in a woman of eighty-seven years. In the latter case the drug was also applied directly to the ulcer for a month. No further recurrence had been observed two years later.

